# Cross sectional analysis of depression amongst Australian rural business owners following cyclone-related flooding

**DOI:** 10.1186/s12995-020-00264-1

**Published:** 2020-05-24

**Authors:** Keersten Cordelia Fitzgerald, Sabrina Winona Pit, Margaret Rolfe, John McKenzie, Veronica Matthews, Jo Longman, Ross Bailie

**Affiliations:** 1 School of Medicine, Western Sydney University, Sydney, Australia; 2 School of Medicine, Western Sydney University, University Centre for Rural Health, Sydney, Australia; 3 The University of Sydney, University Centre for Rural Health, 61 Uralba Street, Lismore, NSW 2480 Australia

**Keywords:** Natural disasters, Mental health, Business, Insurance, Depression, Floods

## Abstract

**Background:**

Flooding is an increasingly prevalent natural hazard worldwide and can have a profound impact on the mental health of those directly and indirectly affected. Little is known about the impact on business owners, who may be particularly vulnerable to the mental health complications of flooding given the additional economic stressors.

**Methods:**

A large cross-sectional survey was conducted six months after severe flooding in the rural Northern Rivers region of New South Wales, Australia in 2017. The survey assessed demographics, probable depression (using the Patient Health Questionnaire-2), flood exposure, flood-related financial factors, prior flood exposure and support from various organisations. Logistic regression was used to identify predictors of probable depression in 653 of the 745 participants who identified as business owners.

**Results:**

The prevalence of probable depression in our sample was 17.0%. A quarter (25.1%) of business owners whose business was flooded suffered from probable depression, compared to 12.4% of non-flooded business owners. The multivariable model for probable depression demonstrated elevated adjusted odds ratios (AOR) for business owners who had to evacuate their business (AOR = 2.11, 95% Confidence Interval (CI) 1.25–3.57) compared to those who did not evacuate. Insurance disputes/rejections were a strong predictor for probable depression (AOR = 3.76, CI 1.86–7.60). Those whose income was reduced due to the flood and had not returned to normal six months post-flood demonstrated an increased AOR for probable depression (AOR 2.53, CI 1.26–5.07) compared to those whose income had returned to normal. The univariable analysis found elevated crude odds ratios (OR) for the cumulative effect of multiple flood exposures and unmet support needs by the state government (OR = 2.74, CI 1.12–6.68). The majority of business owners felt their needs were not met by most organisations providing flood-related support.

**Conclusion:**

The impact of flood exposure and flood-related financial factors on probable depression was highly significant for the business owner population. Furthermore, business owners felt under-supported by flood-related services. These findings highlight the vulnerability of exposed business owners and the need for increased support. Disaster planning programs in conjunction with system level changes such as infrastructure and education are vital for disaster preparedness.

## Background

Flooding is a prevalent and expensive hazard around the world, and in Australia is responsible for the largest economic cost of any natural disaster per annum. These costs consist of damages to infrastructure, agriculture, business and housing, as well as intangible costs in health, social and cultural losses [1]. Floods can have a profound impact on the health of those directly and indirectly affected [2, 3]. These health impacts can include physical injuries, lack of access to healthcare and medications and mental health problems such as post-traumatic stress disorder (PTSD), anxiety and depression [2–4]. Depression is one of the most common mental health consequences of flooding, and some studies show prevalence rates higher than PTSD, up to 35.1% of affected populations [5]. PTSD is less common following natural disasters compared to human-caused disasters according to a 2018 meta-analysis [6]. Furthermore, PTSD-like symptoms, even without meeting the criteria for PTSD diagnosis, can lead to the development of other psychiatric conditions including depression [2]. Therefore due to this striking prevalence, depression can not be ignored as a vital outcome measure that needs to be studied in this context.

Post-disaster, business owners face additional economic pressures as well as the problems which affect the general population [2, 7]. Some of these economic pressures include loss of income, stock and equipment damage, rebuilding issues, insurance matters, loss of electricity and customer and employee shortages [7]. Depressive symptoms are increased in the general population following flood exposure, [2] and we postulate that due to these economic stressors, business owners may be at an increased risk. Therefore, studying this potentially vulnerable group is vitally important to assess mental health outcomes and suggest solutions to address these outcomes. To our knowledge there is no research surrounding the post-flooding mental health of business owners, however there is some limited evidence following natural disasters other than flooding. A Sri Lankan study found that following the 2004 tsunami in Southeast Asia, poor initial mental health was strongly correlated with degree of economic loss. However, mental health recovery was not associated with economic recovery. In this study, mental health was measured using questions related to “return to normalcy” in life and “change in life outlook”, which correlated with the Mental Health Inventory (MHI-5) and the DSM-IV screening questions for PTSD [8]. A Thai study that also looked at the 2004 tsunami found that those who suffered loss of business had significantly poorer mental health outcomes at one year post-tsunami, compared to the unaffected population. Mental health outcomes were assessed in this study using the Short Form Health Survey (SF-36) [9]. However, it is difficult to make direct comparisons from the aforementioned studies due to the different type of natural disaster, the developing country context and the large scale of mortality, morbidity and destruction of the 2004 tsunami.

In light of the complex relationship for business owners between mental health recovery and economic recovery, it suggests that other factors may impact mental health outcomes amongst this group. A number of factors have been identified as having an association with poor mental health outcomes post-flood. Amongst the general population affected by flooding, the degree of flood exposure damage has repeatedly been shown to correlate with poorer mental health. A similar relationship has been found with financial factors such as socioeconomic status and economic losses. On the other hand, both age and gender have yielded inconsistent results [2].

The Northern Rivers region in New South Wales (NSW), Australia, is particularly prone to flooding, with seven major floods documented since 1857 [10]. The most recent event was severe flooding in March 2017 due to heavy rainfall following cyclone Debbie [11]. In Lismore (a large population centre in the region), the levee (constructed in 2005 to protect the Central Business District) was overtopped for the first time, inundating businesses in the town centre [12]. We postulate that this trauma will have had a significant impact on the community and warrants extensive research. Following the March 2017 flooding, to assist business owners, several supports were made available from various sectors including: local volunteer assistance to clean away flood debris; local council and community organisations’ emergency food and shelter assistance; local council and church sponsored business chaplaincy program for emotional and wellbeing support; and state government financial assistance specifically targeted at small business.

This study aims to inform the literature gap surrounding business owners by investigating a number of factors that may influence depression post-flooding in this vulnerable group. These factors include 2017 flood exposures such as business flood and evacuation status, evacuation warning times and home flood status, as well as flood-related financial factors such as insurance disputes/rejections and the flood effect on income. We also investigated factors such as demographics, prior flood exposure, and satisfaction with support received from various organisations post-flood.

## Methods

### Data collection

A cross sectional survey was conducted online and in paper form from September to November 2017 in the Northern Rivers, in North NSW. Recruitment and sample size estimation has been described in detail in a separate paper [13]. The survey was executed in response to flooding in the region in March 2017, six months prior. This survey targeted residents aged 16 years and over, in six local government areas with a total population of approximately 250,000 as of 2018 [14]. Participants were recruited via a snowballing distribution technique, utilising community groups, service providers, local government and local businesses. As the purpose was to investigate relationships between mental health and flooding, rather than to evaluate population prevalence, this purposive sampling recruitment method was appropriate and facilitated the targeting of groups of interest including business owners [13]. Additionally, social media and local media were used to increase survey awareness, and a prize draw with gift vouchers to encourage participation. These techniques were supplemented with door-to-door data collection in randomly selected neighbourhoods in the most affected areas and leaflet drops. Respondents were deemed to have provided consent by returning a questionnaire [13]. The online survey was created using Qualtrics software (version Sept–Nov 2017, Qualtrics Provo Utah).

### Measures

The survey included questions regarding basic demographics, mental health, 2017 flood exposure, flood-related financial factors, prior flood exposure and flood-related support needs.

#### Demographic factors

Demographic factors included age, gender, place of birth, Aboriginal and Torres Strait Islander status, relationship status, education level, current work status, annual household income, and housing status at the time of the flood.

#### Outcome measure: probable depression

The outcome measure, probable depression, was assessed using the Patient Health Questionnaire (PHQ-2), a validated diagnostic tool with scores ranging between 0 and 6. Respondents who scored ≥3 were categorized as having probable depression [15].

#### 2017 Flood exposure

Flood-related factors included business flood status (flooded or not flooded), level of water in the business (not flooded, water in some areas but not all of the business, water below knee in entire business, water between knee and head height in entire business or water above head height in entire business), evacuation status (evacuated or not evacuated) and warning given to evacuate in hours. Non-business flood exposures were also assessed: liveable areas of the home flooded, evacuation status and displacement from home.

#### Flood-related financial factors

Two flood-related financial factors were included: income reduction due to the flood and insurance disputes/rejections despite believing they were fully insured.

#### Previous flood exposure damage

Prior flood exposure included exposure to floods in the Northern Rivers, or damage due to floods in other areas.

#### Flood-related support

Flood-related support needs were assessed by asking whether participants felt their needs were met by the state government, local council, community organisations, insurance companies, emergency services and volunteers/neighbours. Needs were classified as “met” or “unmet” for these variables. Those participants who answered “don’t know” or “not-applicable” were coded as missing.

#### Cumulative effect variables

Cumulative effect variables were derived from existing variables to provide information regarding the impact of compounding exposures on mental health. The “business cumulative effect” variable was calculated by combining business flood status and business evacuation status so that participants were rated as having neither exposure (neither flooded nor evacuated), one exposure (flooded or evacuated) or both exposures (flooded and evacuated). Similarly, a “business and home cumulative effect” variable was calculated by combining business flood status and home flood status.

### Sample

The survey had a total of 2530 responses including business owners and non-business owners. The final sample was composed of respondents who identified themselves as a business owner and who completed the primary outcome measure (probable depression). Farmers were excluded from this group, which left 745 valid responses. Ninety-two responses were excluded due to missing data leaving 653 responses for the univariable and multivariable logistic regression. The samples for the six flood-related support needs variables excluded participants who answered “don’t know” or “not-applicable” leading to samples ranging between 304 and 436 respondents.

### Statistical analyses

The categorical demographic characteristics, 2017 flood exposure factors, flood-related financial factors, prior flood exposure and supports provided were calculated on the sample of 745. Chi square tests of association of these categorical characteristics with the binary outcome of having probable depression were conducted.

Univariable logistic regression models for probable depression were assessed using demographics, 2017 flood exposures and financial factors for participants with complete data (*n* = 653). Any characteristic which was associated with probable depression with a *p* < 0.10 was included in the univariable and multivariable logistic regression models. The model building process was made up of three steps with inclusion of i) demographic factors ii) 2017 flood exposure and iii) financial factors. Variables were included in the final model if they demonstrated an increase in the Nagelkerke R square, demonstrating improved model fit.

The following variables were excluded from the model due to collinearity: current work status (association with household income); business flooding status and water level (association with business evacuation); insurance company support needs (association with insurance disputes/rejections); and home evacuation and displacement (association with liveable area of the home flooded). The association by chi square of these collinear variables is seen in Appendix 1 in Table 7.

Cumulative variables were not included in the multivariable models in favour of the individual variables which comprised them. A second multivariable model is displayed in appendix (Appendix 2 in Table 8) that includes the business cumulative effect variable. Support needs variables were analysed separately, and all were included in univariable logistic analysis. When support factors were included in the multivariable model, after adjusting for demographics, flood exposure and financial factors, none retained significance so were not included in the final logistic model. This is with the exception of the insurance company support needs variable, which was excluded due to collinearity with insurance disputes/rejections. The odds ratios (OR) and adjusted odds ratios (AOR) were reported with 95% confidence intervals. A *p* value < 0.05 was considered to indicate statistical significance, no adjustments were made for multiple comparisons. The statistical program SPSS (Version 25, Armonk, NY: IBM Corp.) was used for data analysis.

### Ethics

Ethics approval was granted by University of Sydney Human Research Ethics Committee and the Aboriginal Health and Medical Research Council Human Research Ethics Committee (Ethics ID 2017/589 and 1294/17 respectively). Completion of questionnaire was taken as informed consent.

## Results

### Demographic characteristics

Of the 745 business owners, 67.2% were female and 46.4% were aged between 35 and 54 years. Gender was associated with probable depression, with 24.6% of male compared to 13.8% of female respondents having probable depression. Aboriginal and Torres Strait Islander status was also associated with probable depression with 56.0% (14/25) of Aboriginal and Torres Strait Islander respondents having probable depression compared to 15.6% (110/705) of non-Indigenous respondents. Similarly, single relationship status, lower education, current work status (unemployment), lower annual household income and housing status at the time of the flood were all found to be associated with probable depression. Age and place of birth were not significantly associated with probable depression (Table 1).

**Table 1:** Demographic characteristics and association with depression amongst business owners (*n* = 745)

Variable	Total		Probable Depression		*P*-value
			*No* *N = 618 (83%)*	*Yes* *N = 127 (17%)*	
	n	%	n (%)	n (%)	
Demographics					
Age (*n* = 733, missing = 12) *Chi-square value = 4.38, df = 2*					
*16–34 years*	95	13	78 (82.1)	17 (17.9)	0.112
*35–54 years*	340	46.4	272 (80)	68 (20)	
*55 years and over*	298	40.7	257 (86.2)	41 (13.8)	
Gender (*n* = 732, missing = 13) *Chi-square value = 13.03, df = 1*					
*Female*	492	67.2	424 (86.2)	68 (13.8)	**< 0.001**
*Male*	240	32.8	181 (75.4)	59 (24.6)	
Place of birth (*n* = 744, missing = 1) *Chi-square value = 0.83, df = 1*					
*Australian born*	618	83.1	509 (82.4)	109 (17.6)	0.362
*Overseas born*	126	16.9	108 (85.7)	18 (14.3)	
Aboriginal and Torres Strait Islander status (*n* = 730, missing = 15) *Chi-square value = 27.94, df = 1*					
*Aboriginal and Torres Strait Islander*	25	3.4	11 (44.0)	14 (56.0)	**< 0.001**
*Non-Indigenous*	705	96.6	595 (84.4)	110 (15.6)	
Relationship status (*n* = 729, missing = 16) *Chi-square value = 30.45, df = 1*					
*Single*	192	26.3	134 (69.8)	58 (30.2)	**< 0.001**
*In a relationship*	537	73.7	469 (87.3)	68 (12.7)	
Highest education (*n* = 725, missing = 20) *Chi-square value = 9.49, df = 2*					
*Year 12 or less*	177	24.4	144 (81.4)	33 (18.6)	**0.009**
*Diploma/Trade/Tafe*	237	32.7	185 (78.1)	52 (21.9)	
*University degree or higher*	311	42.9	273 (87.8)	38 (12.2)	
Current work status (*n* = 736, missing = 9) *Chi-square value = 47.16, df = 2*					
*Not employed*	100	13.6	59 (59.0)	41 (41.0)	**< 0.001**
*Employed*	556	75.5	477 (85.8)	79 (14.2)	
*Retired*	80	10.9	73 (91.3)	7 (8.8)	
Annual household income (*n* = 727, missing = 18) *Chi-square value = 31.87, df = 3*					
*Prefer not to disclose*	110	15.1	92 (83.6)	18 (16.4)	**< 0.001**
*Under $50,000*	242	33.3	178 (73.6)	64 (26.4)	
*$50,000–$100,000*	219	30.1	192 (87.7)	27 (12.3)	
*Over $100,000*	156	21.5	146 (93.6)	10 (6.4)	
Housing status at the time of the flood (*n* = 738, missing = 7) *Chi-square value = 15.46, df = 2*					
*Renting/Other*	202	27.4	150 (74.3)	52 (25.7)	**< 0.001**
*Had a mortgage*	316	42.8	267 (84.5)	49 (15.5)	
*Owned a home outright*	220	29.8	194 (88.2)	26 (11.8)	

### Flood exposure, flood-related financial factors and previous flood exposure

The following 2017 flood exposure factors were found to be significantly associated with probable depression: business flood status, business evacuation, level of flood waters in business, home flood status (liveable areas), home evacuation and any length of displacement from home. The overall prevalence of depression amongst respondents was 17.0%. Of those business owners whose premises were flooded 25.1% reported probable depression, compared to 12.4% of non-flooded business owners. Similarly, 24.5% of those whose businesses were evacuated reported probable depression compared to 12.8% of those who were not evacuated. In contrast, warning time given by emergency services to evacuate business was not associated with probable depression. Both the business cumulative effect, and business and home cumulative effect were significantly associated with probable depression, where increased levels of exposure resulted in higher rates of probable depression. Flood-related financial factors, including insurance disputes/rejections and effect of flood on income, were also strongly associated with probable depression. Prior flood exposure damage appeared not to be associated with probable depression (Table 2).

**Table 2:** Flood exposure, financial factors and previous flood exposure- association with probable depression (*n* = 745)

	Total		Probable Depression		*P* value
			*No* *n = 618 (83%)*	*Yes* *n = 127 (17%)*	
	n	%	n (%)	n (%)	
2017 Flood Exposure					
Business flooded (n = 745, missing = 0) *Chi-square = 19.50, df = 1*					
*Not flooded*	474	63.6	415 (87.6)	59 (12.4)	**< 0.001**
*Flooded*	271	36.4	203 (74.9)	68 (25.1)	
Evacuated business (n = 729, missing = 16) *Chi-square = 16.60, df = 1*					
*Did not have to evacuate*	447	61.3	390 (87.2)	57 (12.8)	**< 0.001**
*Had to evacuate*	282	38.7	213 (75.5)	69 (24.5)	
Level of water in business (n = 738, missing = 7) *Chi-square = 32.72, df = 4*					
*Not flooded*	474	64.2	415 (87.6)	59 (12.4)	**< 0.001**
*Water in some areas but not all of the business*	44	6.0	39 (88.6)	5 (11.4)	
*Water below knee in entire business*	24	3.3	21 (87.5)	3 (12.5)	
*Water between knee and head height in entire business*	120	16.3	86 (71.7)	34 (28.3)	
*Water above head height in entire business*	76	10.3	51 (67.1)	25 (32.9)	
Warning given by emergency services to evacuate business (hours) (*n* = 209, missing = 536) *Chi-square = 0.41, df = 2*					
*No warning*	88	42.1	68 (77.3)	20 (22.7)	0.814
*Four hours or less*	81	38.8	60 (74.1)	21 (25.9)	
*More than four hours*	40	19.1	29 (72.5)	11 (27.5)	
Liveable area of home flooded or damaged (n = 725, missing = 20) *Chi-square = 41.28, df = 1*					
*No*	567	78.2	499 (88.0)	68 (12.0)	**< 0.001**
*Yes*	158	21.8	105 (66.5)	53 (33.5)	
Home evacuation (n = 732, missing = 13) *Chi-square = 52.23, df = 1*					
*No*	608	83.1	534 (87.8)	74 (12.2)	**< 0.001**
*Yes*	124	16.9	76 (61.3)	48 (38.7)	
Any length of displacement from home (n = 745, missing = 0) *Chi-square = 35.66, df = 1*					
*No*	633	85.0	547 (86.4)	86 (13.6)	**< 0.001**
*Yes*	112	15.0	71 (63.4)	41 (36.6)	
Cumulative business impact (n = 729, missing = 16) *Chi-square = 24.22, df = 2*					
*Business neither flooded nor evacuated*	379	52.0	334 (88.1)	45 (11.9)	**< 0.001**
*Business flooded or evacuated*	151	20.7	126 (83.4)	25 (16.6)	
*Business flooded and evacuated*	199	27.3	143 (71.9)	56 (28.1)	
Cumulative business and home impact (n = 725, missing = 20) *Chi-square = 50.06, df = 2*					
*Neither business nor home flooded*	381	52.6	350 (91.9)	31 (8.1)	**< 0.001**
*Business or home flooded*	271	37.4	208 (76.8)	63 (23.2)	
*Business and home flooded*	73	10.1	46 (63.0)	27 (37.0)	
Flood-Related Financial Factors					
Insurance disputes/rejections (n = 738, missing = 7) *Chi-square = 66.24, df = 1*					
*No*	669	90.7	579 (86.5)	90 (13.5)	**< 0.001**
*Yes*	69	9.3	33 (47.8)	36 (52.2)	
Flood effect on income (*n* = 738, missing = 7) *Chi-square = 35.73, df = 2*					
*No effect*	431	58.4	375 (87.0)	56 (13.0)	**< 0.001**
*Income reduced after flood, but now back to normal*	182	24.7	157 (86.3)	25 (13.7)	
*Income remains reduced*	125	16.9	81 (64.8)	44 (35.2)	
Previous Flood Exposure Damage (prior to 2017)					
Previous damage to home/work due to flood (n = 738, missing = 7) *Chi-square = 2.93, df = 2*					
*No*	284	38.5	228 (80.3)	56 (19.7)	0.231
*Once-twice*	235	31.8	202 (86.0)	33 (14.0)	
*Several times*	219	29.7	182 (83.1)	37 (16.9)	
Previous exposure to Northern Rivers flood (n = 744, missing = 1) *Chi-square = 0.83, df = 2*					
*No*	101	13.6	83 (82.2)	18 (17.8)	0.659
*Once-twice*	238	32.0	194 (81.5)	44 (18.5)	
*Several times*	405	54.4	341 (84.2)	64 (15.8)	

### Perceived support needs

Table 3 demonstrates the perception of flood-related support needs. Overall, business owners were most likely to feel their needs were met by volunteers/neighbours (56%), followed by community organisations (39.1%) and emergency services (36.2%). However, a large a number of participants answered “don’t know” or “not-applicable” to these questions and were therefore excluded from data analysis presented in Table 3. The odds for probable depression in those with unmet needs was always higher than that of met needs, but was only statistically significant for support from State Government or insurance companies. Business owners who felt that the state government or insurance company did not meet their needs had almost three times the odds of having probable depression (OR = 2.74, CI 1.12–6.68 and OR = 2.78, CI 1.26–6.13 respectively) compared to people who thought their needs had been met.

**Table 3:** Probable depression association and crude ORs for support factors: univariable logistic regression (*n* = 745)

	Total		Probable Depression		*P*-value	Probable Depression OR(95% CI)	*P*-value
			*No* *n = 618 (83%)*	*Yes* *n = 127 (17%)*			
	n	%	n (%)	n (%)			
Perceived Support Needs by:							
State government (*n* = 335)^a^ *Chi-square = 5.26, df = 1*							
*Needs unmet*	284	84.8	208 (73.2)	76 (26.8)	**0.022**	2.74 (1.12–6.68)	**0.027**
*Needs met*	51	15.2	45 (88.2)	6 (11.8)		REF	
Local council (*n* = 394)^b^ *Chi-square = 3.39, df = 1*							
*Needs unmet*	300	76.1	221 (73.7)	79 (26.3)	0.066	1.74 (0.96–3.16)	0.068
*Needs met*	94	23.9	78 (83.0)	16 (17.0)		REF	
Community organisations (n = 335)^c^ *Chi-square = 2.89, df = 1*							
*Needs unmet*	204	60.9	145 (71.1)	59 (28.9)	0.089	1.57 (0.93–2.64)	0.091
*Needs met*	131	39.1	104 (79.4)	27 (20.6)		REF	
Insurance company (*n* = 304)^d^ *Chi-square = 6.78, df = 1*							
*Needs unmet*	244	80.3	171 (70.1)	73 (29.9)	**0.009**	2.78 (1.26–6.13)	**0.012**
*Needs met*	60	19.7	52 (86.7)	8 (13.3)		REF	
Emergency services (*n* = 323)^e^ *Chi-square = 1.37, df = 1*							
*Needs unmet*	206	63.8	148 (71.8)	58 (28.2)	0.243	1.37 (0.81–2.33)	0.244
*Needs met*	117	36.2	91 (77.8)	26 (22.2)		REF	
Volunteers/Neighbours (*n* = 436)^f^ *Chi-square = 0.99, df = 1*							
*Needs unmet*	192	44.0	145 (75.5)	47 (24.5)	0.320	1.26 (0.80–1.98)	0.321
*Needs met*	244	56.0	194 (79.5)	50 (20.5)		REF	

*REF* Reference category

*Missing/Don’t Know/Not Applicable:*^*a*^*n = 410;*^*b*^*n = 351;*^*c*^*n = 410;*^*d*^*n = 441;*^*e*^*n = 422;*^*f*^*n = 309*

### Univariable logistic regression

Table 4 presents the univariable logistic regression results for the demographic factors. The following factors all demonstrated increased OR for probable depression: respondents who were male, Aboriginal and Torres Strait Islander, single, those who are currently unemployed, those with a household income of <$50,000 per annum and people who were renters at the time of the flood.

**Table 4:** Probable depression crude ORs for demographics: univariable logistic regression (*n* = 653)

	Probable Depression OR (95% CI)	*P* value
Demographics		
Gender (missing = 0)		
*Female*	REF	
*Male*	1.88 (1.24–2.86)	**0.003**
Aboriginal and Torres Strait Islander status (missing = 0)		
*Aboriginal and Torres Strait Islander*	7.97 (3.44–18.47)	**< 0.001**
*Non-Indigenous*	REF	
Relationship status (missing = 0)		
*In a relationship/Married*	REF	
*Single/Divorced/Separated/Widowed*	3.31 (2.15–5.09)	**< 0.001**
Education (*n* = 644, missing = 9)		0.069
*Year 12 or less*	REF	
*Diploma/Trade/Tafe*	1.26 (0.73–2.17)	0.406
*University degree or higher*	0.71 (0.41–1.23)	0.221
Current work status (*n* = 646, missing = 7)		**< 0.001**
*Not employed*	4.15 (2.48–6.95)	**< 0.001**
*Employed*	REF	
*Retired*	0.57 (0.24–1.37)	0.210
Annual household income (missing = 0)		**< 0.001**
*Prefer not to disclose*	2.76 (1.14–6.66)	**0.024**
*Under $50,000*	6.37 (3.05–13.31)	**< 0.001**
*$50,000–$100,000*	2.20 (0.99–4.89)	0.052
*Over $100,000*	REF	
Housing status at the time of the flood (missing = 0)		**< 0.001**
*Renting/Other*	3.03 (1.71–5.38)	**< 0.001**
*Had a mortgage*	1.49 (0.85–2.62)	0.168
*Owned a home outright*	REF	

*REF* Reference category

Table 5 presents the univariable logistic regression for the 2017 flood exposure factors, including cumulative variables, and flood-related financial factors. People’s whose businesses had been flooded or whose business had been evacuated had more than twice the odds of probable depression compared to business owners who were unaffected. The severity of flood exposure within the business premises (measured by the level of flood waters in the business) was associated with increasing levels of probable depression. In terms of cumulative effects, business owners who were both flooded and evacuated from their business had an increased OR (OR = 2.98, CI 1.85–4.81), and there was a trend towards significance for those with only one of these exposures (OR = 1.61, CI 0.93–2.80), compared to those who had neither. Business and home cumulative effect included business flood status and home flood status, both those with one exposure (OR = 3.63 CI 2.23–5.92) and both exposures (OR = 6.47 CI 3.41–12.28) showed significantly increased ORs compared to those who had neither. Insurance dispute or rejection was one of the strongest predictors for increased probable depression (OR = 4.99, CI 2.81–8.88). If income was reduced after the flood but had returned to normal within six months, the OR was not significantly different from those whose income was not affected by the flood. However, if the income had been reduced by the flood and remained reduced, there was an increased OR compared to those whose income had returned to normal (OR = 3.71, CI 2.02–6.78).

**Table 5:** Probable depression crude ORs for flood exposure and financial factors: univariable logistic regression (*n* = 653)

	Probable Depression OR (95% CI)	*P* value
2017 Flood Exposure		
Business flooded (missing = 0)		
*Not flooded*	REF	
*Flooded*	2.39 (1.57–3.63)	**< 0.001**
Evacuated business (missing = 0)		
*No*	REF	
*Yes*	2.22 (1.46–3.37)	**< 0.001**
Degree of flooding to business (n = 646, missing = 7)		**< 0.001**
*Not flooded*	REF	
*Water in some areas but not all*	0.61 (0.18–2.06)	0.428
*Water below knee in entire business*	0.73 (0.17–3.24)	0.638
*Water between knee and head height in entire business*	2.90 (1.71–4.91)	**< 0.001**
*Water above head height in entire business*	3.92 (2.20–6.97)	**< 0.001**
Liveable area of home flooded or damaged (missing = 0)		
*No*	REF	
*Yes*	3.87 (2.47–6.05)	**< 0.001**
Home evacuation (*n* = 649, missing = 4)		
*No*	REF	
*Yes*	5.08 (3.15–8.20)	**< 0.001**
Any length of displacement from home (missing = 0)		
*No*	REF	
*Yes*	4.45 (2.70–7.32)	**< 0.001**
Business cumulative effect (missing = 0)		**< 0.001**
*Business neither flooded nor evacuated*	REF	
*Business flooded or evacuated*	1.61 (0.93–2.80)	0.092
*Business flooded and evacuated*	2.98 (1.85–4.81)	**< 0.001**
Business and home cumulative effect (missing = 0)		**< 0.001**
*Neither business nor home flooded*	REF	
*Business or home flooded*	3.63 (2.23–5.92)	**< 0.001**
*Business and home flooded*	6.47 (3.41–12.28)	**< 0.001**
Flood-Related Financial Factors		
Insurance disputes/rejections (missing = 0)		
*No*	REF	
*Yes*	4.99 (2.81–8.88)	**< 0.001**
Flood effect on income (missing = 0)		**< 0.001**
*No effect*	0.92 (0.54–1.59)	0.773
*Income reduced after flood, but now back to normal*	REF	
*Income remains reduced*	3.71 (2.02–6.78)	**< 0.001**

*REF* Reference category

### Multivariable logistic regression

For the multivariable logistic model, after adjusting for demographic and flood related factors, the OR for business evacuation remained similar to that of the univariable model AOR = 2.11, CI 1.25–3.57, compared to OR = 2.22 (Tables 5 and 6). However, the AOR for home flooding or damage, although remaining significant, reduced from OR = 3.87 to AOR = 2.14, CI 1.25–3.66, compared to those who were not affected. Insurance disputes/rejections remained one of the strongest predictors for probable depression (AOR = 3.76, CI 1.86–7.60, down from OR = 4.99). Prolonged reduction in income also had a significantly increased AOR 2.53 (1.26–5.07). In summary, flood exposure and financial factors were associated with probable depression after adjustment for relevant demographic variables.

**Table 6:** Probable depression AOR amongst business owners: multivariable logistic regression Model (*n* = 653)

	Probable Depression AOR(95% CI)	*P* value
Demographics		
Gender		
*Male*	2.44 (1.47–4.04)	**0.001**
*Female*	REF	
Aboriginal and Torres Strait Islander status		
*Aboriginal and Torres Strait Islander*	6.48 (2.43–17.25)	**< 0.001**
*Non-Indigenous*	REF	
Relationship status		
*In a relationship/Married*	REF	
*Single/Divorced/Separated/Widowed*	2.22 (1.30–3.80)	**0.004**
Annual household income		**0.013**
*Prefer not to disclose*	1.66 (0.62–4.47)	0.318
*Under $50,000*	3.42 (1.47–7.98)	**0.004**
*$50,000–$100,000*	1.71 (0.73–3.99)	0.218
*Over $100,000*	REF	
Housing status at the time of the flood		**0.013**
*Renting/Other*	2.73 (1.40–5.33)	**0.003**
*Had a mortgage*	1.89 (0.97–3.70)	0.062
*Owned a home outright*	REF	
2017 Flood Exposure		
Had to evacuate business		
*No*	REF	
*Yes*	2.11 (1.25–3.57)	**0.005**
At least one liveable area of home flooded or damaged		
*No*	REF	
*Yes*	2.14 (1.25–3.66)	**0.006**
Flood-related Financial Factors		
Insurance disputes/rejections		
*No*	REF	
*Yes*	3.76 (1.86–7.60)	**< 0.001**
Flood effect on income		**0.030**
*No effect*	1.41 (0.75–2.68)	0.289
*Income reduced after flood, but now back to normal*	REF	
*Income reduced after flood and remains reduced*	2.53 (1.26–5.07)	**0.009**

*REF* Reference Category

Nagelkerke R square = 0.329

## Discussion

Our results identified multiple factors that influenced the likelihood of probable depression amongst business owners post-flooding. Key findings were that those who had their business flooded or evacuated, those who had insurance disputes/rejections or those whose income was persistently reduced six months post-flood had an increased risk for probable depression. The majority of business owners felt their needs were not met by most organisations providing flood-related support. We identified that in our sample 25.1% of business owners whose businesses were flooded reported probable depression, compared to 12.4% of those whose businesses were not flooded. This compares to the Australian national 12 month prevalence of depression of 4.1% [16]. Rates of depression post flooding vary greatly between studies, but have been as high as 35.1% in affected populations [5].

### Business flooding and the impact on income

Business flood exposure put business owners at an increased risk of probable depression and this was markedly higher with increasing depths of water inundation. Similarly, a Thai study reported that those who suffered a loss of business as a result of the 2004 tsunami had poorer overall mental health scores one-year post-tsunami [9]. This may be explained by the direct and indirect economic impacts that contribute to economic vulnerability of small businesses post-disaster and may act as significant stressors. The economic impacts can result from physical injury, direct damage to the business premises, evacuation, associated loss of income and loss of stock/equipment and associated repairs. Indirect impacts include lack of customers and staff, issues with support post event, supply chain interruption and difficulties with transport and access [7, 17]. Furthermore, other factors that affect the broader working community may act as additional stressors for business owners. For example, as identified in a study of American university students, flood-related work disruption resulted in poorer self-reported mental health status. Factors that influence this may include communication difficulties between employees and employers, lack of access to social support at work, uncertainty regarding flood-related work expectations and difficulty travelling to work [18]. A 2019 Bangladeshi study found that being younger, earning an income, having physical injuries due to a disaster, and post-disaster work absenteeism were risk factors of depression post-cyclone Mora [19]. Unemployment and job insecurity, independent of a natural disaster, have also been repeatedly shown to be associated with depression all around the world [19, 20]. It has also been found that unemployment remains a significant risk factor for depression independent of other contributing factors such as social support, financial stress and a sense of personal control [20].

Our study found that persistent reduction in income due to the floods was a significant risk factor for probable depression (AOR = 2.53, 1.26–5.07). Whereas, initial reduction in income that had returned to normal within six months had no effect on probable depression. In the general population, an association between financial losses and poor mental health outcomes has been established, [2] however ongoing economic recovery may be more complex for business owners due to additional financial stressors. Unlike our results, a Sri Lankan study following the 2004 tsunami, found little association between economic recovery and mental health recovery for affected business owners. However, as discussed earlier, it is difficult to directly compare this study with ours [8].

Our findings provide evidence that supporting businesses after the disaster and assisting the local economy in its recovery could help to reduce the mental health burden on the business community, which may in turn help the broader community. Indeed, post-flood, a chaplaincy program was implemented by the local government in the Lismore area to assist business owners with emotional and psychological support. This program was largely well received by business owners, and is credited to have both provided psychological support, as well as raise mental health awareness in the community [21]. Furthermore, addressing economic vulnerability of business owners prior to a flood in disaster prone areas may assist in preventing adverse consequences.

### Business evacuation and evacuation warning time

Business evacuation was significantly associated with probable depression. Home evacuation has been found to be associated with poorer mental health in previous research, however this has not yet been shown in business evacuation [2, 22]. We found that business evacuation warning time was not significantly related to probable depression. The research surrounding this topic is somewhat conflicting. A 2007 English study found that the economic benefit of flood warnings is low for households because portable items consist of a low proportion of overall household property [23]. This may not be the case for business owners, especially in industries with large amounts of moveable stock. The aforementioned study also found that simply receiving a warning did not improve mental health outcomes, but if those who did not receive a warning were excluded, there was a small benefit to longer warning times [23]. Our study was not consistent with these findings. Further research may be necessary to determine the entire scope of benefits of early evacuation warnings, including physical and mental health and economic preparedness for business owners.

### Insurance

Insurance disputes and rejections (including home, business or other property/possessions) affected 9.3% of all business owners who thought they were fully insured and was one of the strongest associations with probable depression. Insurance problems post-flooding has been repeatedly shown to result in poorer mental health in the general population [2, 22, 24, 25]. Business owners may be particularly vulnerable to insurance disputes because it can limit their access to capital necessary to reopen their business and thereby contribute to long-term loss of income.

A 2018 study found that 56% of businesses in one area of our study region did not have flood insurance and 31% were unsure if they had flood insurance [26]. Exorbitant cost has been identified as a perceived barrier to flood insurance by business owners [11, 26]. There is significant capacity for improvement in the insurance process including affordability, speed and ease of claiming, being transparent regarding inclusions and exclusions, and improving communication [11].. Such changes may have a positive impact on business owner’s mental health in the event of future flooding.

### Prior flood exposure

Prior flood exposure was not significantly associated with probable depression in our study. However, in the general population, there is some evidence to suggest that prior flood exposure may increase the risk of mental health problems including depression [5, 27]. This may be explained by the cumulative effect of repeated flood exposure particularly in vulnerable groups and fear of future flooding based on prior negative experiences [5].

We postulate that prior flood exposure may be a complex factor for business owners as prior flood exposure has been shown to improve flood preparedness. Therefore, flood preparedness may improve the economic and mental health outcomes for business owners [28]. These two conflicting factors, repeated trauma and flood preparedness, may be contributing to the lack of significance of prior flood exposure in our study. Further research in this area is warranted based on the conflicting results with prior literature.

### Satisfaction with support services post-flood

Probable depression was more prevalent in those who felt their flood-related support needs were unmet. However, this was not found to be statistically significant for most organisations, after adjusting for other factors, with the exception of insurance companies. Business owners were most likely to have felt that their needs were met by volunteers and neighbours. This finding encourages support of volunteers in both the pre- and post-event response. Aside from volunteers and neighbours, less than 50% of respondents were satisfied with the flood response of any service/organisation. This suggests that significant improvement is required by these organisations to increase satisfaction of the affected population. It is common for people to feel isolated from authorities after a flood, [24] and flood victims are more likely to come forward to trusted members of the community rather than mental health professionals [2] This should be used guide the implementation of post-flood programs, such as was seen with the church sponsored Lismore Chaplaincy program.

### Cumulative flood exposure

The negative impact of flooding, evacuation and displacement from home is well established in the literature and our findings were in concordance with this [2, 5, 22, 24]. We derived two innovative cumulative indices. The business cumulative effect index looked at business flooding in combination with business evacuation. The business and home cumulative variable combined business and home flood status. Both were associated with higher rates of probable depression amongst business owners. Our findings demonstrate that those with compounding exposures are particularly vulnerable and should be identified as a target for mental health and economic support.

### Demographic factors

Predictive demographic factors of probable depression included male gender, Aboriginal or Torres Strait Islander, being single, low household income and renting at the time of the flood. Without knowledge of pre-existing depression rates it is difficult to assess these factors in a cross-sectional study. However, low income and renting have previously been associated with increased mental health vulnerability post-flooding [22]. Financial pressure pre-disaster may mean a business owner was particularly impacted by an interruption to income and the associated stressors of flooding.

Our study identified that male business owners had twice the AOR of probable depression compared to females. Prior research has found either no association with gender or that females have poorer mental health outcomes after flooding [2, 22]. This requires further investigation.

The highest AOR (6.48) for probable depression was in the Aboriginal and Torres Strait Islander population, however the sample size was small (*n* = 24). In general, the Aboriginal and Torres Strait Islander population have an increased mental health disease burden a result of continuing discrimination, social and economic inequality stemming from colonisation and disempowerment, [29] and may be particularly susceptible to external stressors such as flooding.

### Disaster preparedness

A crisis management framework consisting of disaster prevention, response and recovery, [7] can contribute to minimising the impact of flooding events on business owners. Given the link we have established between economic recovery and mental health recovery, flood preparedness is paramount in minimising the initial impacts on business owners. Individual prevention measures may include disaster plans and flood insurance. On a broader scale, flood infrastructure, such as levees, can also assist in mitigating the impact of floods. For example, a levee was recently built in the study region, prior to the March 2017 flood. However, it is proposed that the relatively new levee provided a false sense of security amongst the local business community, especially amongst those without prior flood experience, and it took many by surprise when it overflowed [26]. It has been suggested that further flood education by the local government may have prevented the lack of preparedness in the study community [11, 26]. In summary, individual disaster plans in conjunction with system level changes such as infrastructure and education are vital for disaster preparedness.

### Limitations

One limitation of this study is that the sample is not representative of the general population [13]. Self-selection bias would inevitably have favoured those who had been affected by the flooding. Furthermore, the survey relied on self-reported data which may affect the accuracy of the information. However, the survey used validated instruments wherever possible (including the PHQ-2 for probable depression) and the methods were in line with previous literature [2, 22]. Although, PHQ-2 is a brief, well-known and validated tool, the longer versions may have increased the accuracy of measuring depressive symptoms. However, the authors chose the shortest measure to enable higher response and completion rates and thus have more meaningful data, and took into account survey length, the inconvenience and potential aversion towards survey completion and survey fatigue.

Secondly, business owners self-identified and the survey did not include the industry groups, therefore we cannot provide a distribution within the business owner sample. Furthermore, business owners may also have been affected by the flood in other areas of their life, such as their home and loved ones. This is difficult to separate and may have an impact on the outcome results. For this reason, home flooding status was adjusted for in the final model. Lastly, given that the pre-flood prevalence of probable depression amongst our population group is not known, we instead compared exposed business owners to non-exposed business owners.

## Conclusion

The association of probable depression with flood exposure and flood-related financial factors was highly significant amongst business owners. Business flooding, evacuation, high levels of water inundation, insurance disputes and persistent reduction in income, were all important predictive factors. Cumulative impacts of both business and home factors also proved to be significant. These findings highlight the vulnerability of exposed business owners and the need for more effective support. The poor satisfaction of business owners with flood-related services confirms the need for improvement. Improvements may include individual disaster planning programs in conjunction with system level changes such as infrastructure and education which are vital for disaster preparedness.

## Data Availability

The datasets supporting the conclusions of this article are not available form the corresponding author due to the sensitive nature of the data and the consent being provided for participation in the specific study.

## Appendix 1

**Table 7:** Chi-Square analysis of collinear variables

Variable Included in the Model	Variable Excluded from the Model	Chi-Square	
		df	*p*-value
Annual household income	Current work status	6	**< 0.001**
Business evacuation	Business flooded	1	**< 0.001**
Business evacuation	Level of water in business	5	**< 0.001**
Liveable area of home flooded	Home evacuation	1	**< 0.001**
Liveable area of home flooded	Displaced from home	1	**< 0.001**
Believed they were fully insured but the insurance company rejected or disputed their claim	Insurance company support needs	1	**< 0.001**

## Appendix 2

**Table 8:** Probable depression AOR amongst business owners: multivariable logistic regression model including cumulative business exposure (*n* = 653)

	Probable Depression AOR(95% CI)	*P* value
Demographics		
Gender		
*Male*	2.34 (1.41–3.89)	**0.001**
*Female*	REF	
Aboriginal and Torres Strait Islander status		
*Aboriginal and Torres Strait Islander*	6.40 (2.39–17.11)	**< 0.001**
*Non-Indigenous*	REF	
Relationship status		
*In a relationship/Married*	REF	
*Single/Divorced/Separated/Widowed*	2.24 (1.31–3.84)	**0.003**
Annual household Income		**0.012**
*Prefer not to disclose*	1.56 (0.58–4.19)	0.380
*Under $50,000*	3.38 (1.46–7.86)	**0.005**
*$50,000–$100,000*	1.68 (0.72–3.93)	0.230
*Over $100,000*	REF	
Housing status at the time of the flood		**0.008**
*Renting/Other*	2.89 (1.47–5.65)	**0.002**
*Had a mortgage*	1.90 (0.98–3.72)	0.059
*Owned a home outright*	REF	
2017 Flood Exposure		
Cumulative business exposure		**0.013**
*Business neither flooded nor evacuated*	REF	
*Business flooded or evacuated*	1.80 (0.94–3.46)	0.079
*Business flooded and evacuated*	2.63 (1.38–5.04)	**0.003**
At least one liveable area of home flooded or damaged		
*No*	REF	
*Yes*	2.14 (1.25–3.67)	**0.005**
Flood-Related Financial Factors		
Believed they were fully insured but the insurance company rejected or disputed their claim		
*No*	REF	
*Yes*	3.50 (1.73–7.10)	**0.001**
Flood effect on income		**0.044**
*No effect*	1.58 (0.82–3.06)	0.171
*Income reduced after flood, but now back to normal*	REF	
*Income reduced after flood and remains reduced*	2.43 (1.21–4.88)	**0.013**

*REF* Reference Category

Nagelkerke R square = 0.331

## Abbreviations

PTSD: Post-Traumatic Stress Disorder
NSW: New South Wales
PHQ-2: Patient Health Questionnaire
OR: Odds Ratio
AOR: Adjusted Odds Ratio
CI: 95% Confidence Interval
REF: Reference category
